# Low-Stress and Optimum Design of Boost Converter for Renewable Energy Systems

**DOI:** 10.3390/mi13071085

**Published:** 2022-07-08

**Authors:** Kashmala Salim, Muhammad Asif, Farman Ali, Ammar Armghan, Nasim Ullah, Al-Sharef Mohammad, Ahmad Aziz Al Ahmadi

**Affiliations:** 1 Department of Electrical Engineering, Qurtuba University of Science and IT, Dera Ismail Khan 29050, Pakistan; kashmalasaleem@qurtuba.edu.pk (K.S.); drfarmanali.optics@qurtuba.edu.pk (F.A.); 2 Department of Electrical Engineering, Main Campus, University of Science & Technology, Bannu 28100, Pakistan; 3 Department of Electrical Engineering, College of Engineering, Jouf University, Sakaka 72388, Saudi Arabia; aarmghan@ju.edu.sa; 4 Department of Electrical Engineering, College of Engineering, Taif University, Al-Hawiyah P.O. Box 888, Saudi Arabia; m.alsharef@tu.edu.sa (A.-S.M.); aziz@tu.edu.sa (A.A.A.A.)

**Keywords:** DC–DC converters, Flyback boost converter using Si-MOSFET switch technology, Flyback boost converter using GaN-HEMT switch technology

## Abstract

This paper examines the design and analysis of DC–DC converters for high-power and low-voltage applications such as renewable energy sources (RESs) and comparisons between converters based on switch stresses and efficiency. The RESs including photovoltaic arrays and fuel cell stacks must have enhanced output voltages, such as 380 V DC in the case of a full bridge inverter or 760 V DC in the case of a half bridge inverter, in order to interface with the 220 V AC grid-connected power system. One of the primary difficulties in developing renewable energy systems is enhancing DC–DC converters’ efficiency to enable high step-up voltage conversion with high efficiency and low voltage stress. In the present work, the efficiency, current, and voltage stress of switches of an isolated Flyback boost converter, simple DC–DC Boost converter, and an Interleaved boost converter, are explored and studied relatively. The most suitable and optimized options with a high efficiency and low switching stress are investigated. The more suitable topology is designed and analyzed for the switch technology based on the Silicon-Metal Oxide Semiconductor Field Effect Transistor (Si-MOSFET) and the Gallium Nitride-High Electron Mobility Transistor (GaN-HEMT). The Analytical approach is analyzed in this paper based on efficiency and switching stress. It is explored that GaN HEMT based Flyback boost converter is the best. Finally, the future direction for further improving the efficiency of the proposed boost converter is investigated.

## 1. Introduction

**Figure 1:**
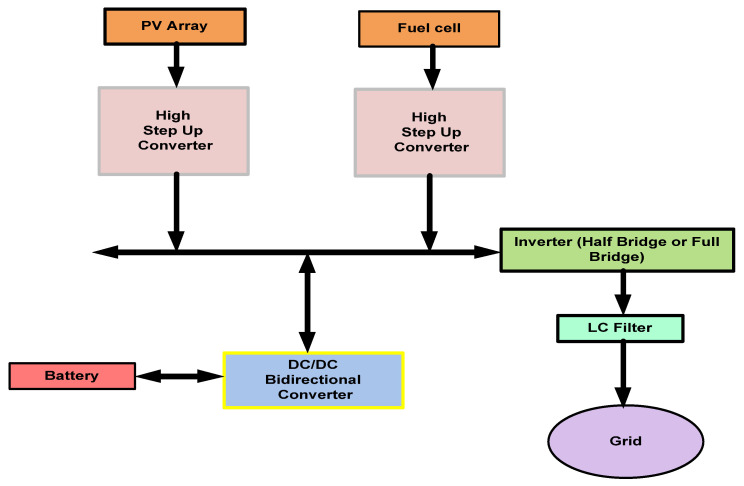
Block diagram of a hybrid renewable energy grid-connected system.

Recently, there has been a continuous increase in environmental issues. As a result, retardations have occurred in the power generation based on fossil fuel [1,2]. In addition, the rapid growth of energy consumption across the globe has increased numerous renewable energy-based power generation systems, control algorithms, smart grids, advanced energy conversion, hybrid energy storage systems, and management systems. These systems require advanced power converters to convert the voltage efficiently; as a result, the power generation based on renewable energy sources will be enhanced [3,4]. Therefore, recently, power generation through improved control techniques and better power switching devices is one of the hot research areas [5,6]. One of the main concerns is that renewable energy sources have a low Direct Current (DC) output voltage, such as fuel cell stacks and photovoltaic arrays. It is important to increase the range of voltage to the next level, e.g., 380 V DC in the case of the inverter (full bridge) and 760.00 V-DC in the case of the inverter (half bridge) for yielding 220 V AC output to be interfaced with a grid-connected power system of 220 V AC [7]. DC-to-DC converter development for delivering a high step-up voltage conversion with a low switch stress and high efficiency is one of the key challenges in advancing renewable energy systems [8]. Figure 1 shows the block diagram of a single-phase hybrid renewable energy grid-connected system. In general, such a system consists of a photovoltaic (PV) array, fuel cell stack, batteries, DC-to-DC converters, and inverter [9]. Those DC-to-DC converters with high step-up conversion ratios are needed to interface low-voltage outputs of fuel cell stacks and photovoltaic arrays, typically 22–48 V, with a relatively high-voltage (200+ V) DC link for supplying standalone or grid-connected AC loads through an inverter [10]. 

To assist this growth trend and attain the required performance of the system, the development of enhanced power converters with improved control algorithms and switching devices is essential. Furthermore, the need for communication capability, improvement in the power level, and performance of the converters system are also assessed to have a rapid growth to assist enhancement in the present technologies. Therefore, to realize the above aims, it is essential to consider advanced semiconductor switching features, e.g., lower switching losses, high switching frequency, smaller sizes, thermal capability, and higher power densities.

To obtain the above stated quality standards and application goals, gallium nitride (GaN)-based High Electron Mobility Transistor (HEMT) (wide-bandgap) and Silicon-Metal Oxide Semiconductor Field Effect Transistor (SiC-based MOSFET) are commonly referred to in this paper as the best switching devices [11,12,13,14]. This research focuses on studying several DC-to-DC boost converters in terms of their suitability to be used in such power systems. 

### Related Work

Researchers have examined various converters to discover converters that give greater voltage conversion ratios with a step-up property, higher efficiency, and lower stresses of switch voltage. There are numerous DC–DC boost converters in the market; however, by reasons of high cost and complex structures, unnecessary auxiliary circuits, parasitic ringing issues, and high power consumptions, these converters cannot be recommended for renewable energy systems. The type of isolated converters can upsurge the system’s structure and expense while increasing losses. [15] proposes a novel interleaved boost converter for Uninterruptible Power Supply (UPS) applications having a high Voltage Conversion Ratio (VCR). The converter employs a magnetically connected circuit similar to that of a traditional converter. Reference [16] proposes a converter with a single stage for a power generation system based on the PV. This work has explored the importance of such a converter when a lower efficiency and reliability, higher dimensions and cost, and parts are the main targets. This type of converter enhances the solar arrays’ relatively low voltage and converts input DC electricity to generate AC power with better quality to be faded to the grid. In this case, the highest quantity of power will be taken out from the PV array. In [17], the authors have suggested a stage-shift-controlled three-stage, transformer-secluded DC-to-DC converter for fuel cells with lower voltages. On the source side, the converter greatly raises the voltage. It runs at 240 amperes, creating the strategy of a DC–DC converter with a higher current and lower voltage extremely difficult, reduced transformer turns ratio by keeping the voltage constant and decreasing dimensions of inactive components, e.g., the filter capacitor that is used at the output and bus capacitor that is used at the input. Normally the above concept is applied in three interleaving phases for organizing loads, eradicating the ripple current of the inductor at angles having a shift of larger than one hundred twenty degrees and Zero-Voltage–Zero-Current (ZVZC) switches. In [18,19,20], a group of DC-to-DC converters is discussed with a simple topology having high efficiencies. The suggested converters based on coupled winding and diode perform better than their counterparts, i.e., active-clamp. By recycling leakage energy and alleviating the reverse recovery issue, better efficiency is achieved. Wide bandgap devices are game-changing power electronics technology for high-efficiency and high-frequency applications. In [21], the authors have discussed a DC-to-DC boost converter topology that integrates the properties of the interleaved set up. For this purpose, a 100 W prototype converter is suggested with a PIC16F microcontroller. 

For low- and high-output voltage applications, a DC-to-DC boost converter topology was presented in [22] using two hybrid voltage multiple cells, a single switch, and three winding couple inductors. In [23], the authors discussed a hybrid non-isolated DC–DC commutation cell and derived three different DC–DC converters, a buck-type, a boost-type, and a buck-boost-type, which is generated by the integration between a conventional commutation cell and ladder-type passive switched capacitor (SC). A cell 1 kW prototype was developed to verify the operation of the three proposed converters. To maintain the output voltage of DC-to-DC inverting buck boost power converter operation, the authors have presented a novel Lyapunov function=based robust nonlinear proportional integral controller in [24]. The paper [25], discusses the quadratic Lyapunov function for the classical two-level converters and two switching control designing. 

## 2. Proposed and Analytical Modeling

**Figure 2:**
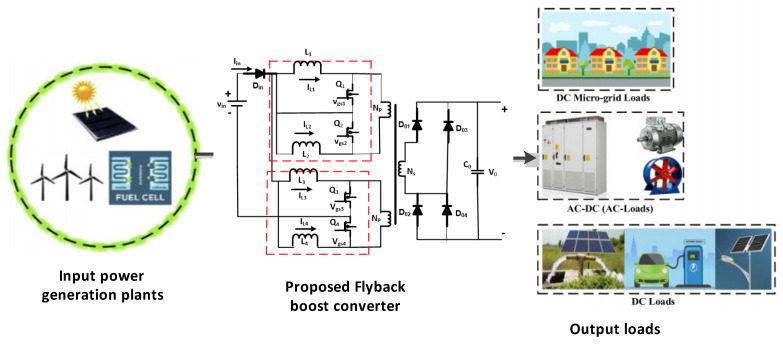
Proposed Flyback-based converter.

The performance and investigation of DC–DC converters, i.e., interleaved boost converter, Flyback-based converter, and simple boost converter, are briefly demonstrated as shown in Figure 2. The analysis of DC–DC converters means to derive expressions for the inductor currents, output, and capacitor voltages of the targeted converters by applying two different techniques, i.e., capacitor charge balance and inductor volt–second balance [26]. Flyback-based and interleaved boost converters were analyzed for different switching modes. Two input levels, an associated transformer, current-fed input circuits, and a subsidiary rectifier bridge make up the converter. Each input-stage course has two control switches (power) and two inductors. To allow for the overlapping process, the values of Q1 and Q4 switches should be higher than 0.5 in order to achieve smooth conversions. These can be mathematically expressed [27] as the following.

(1) $$\phi =D\;Ts-\frac{Ts}{2}$$

(2) $$\phi =\left(2D-1\right)\;\frac{Ts}{2}$$

In the above equations, D is duty ratio, ϕ is the phase of the waveform, and Ts is the period of switching.

Actually, D denotes the Pulse Width Modulation (PWM) generator’s duty cycle, whereas Ts denotes the switching period. There are always four operational modes during the switching time.

### 2.1. First Mode (t0–t1)

**Figure 3:**
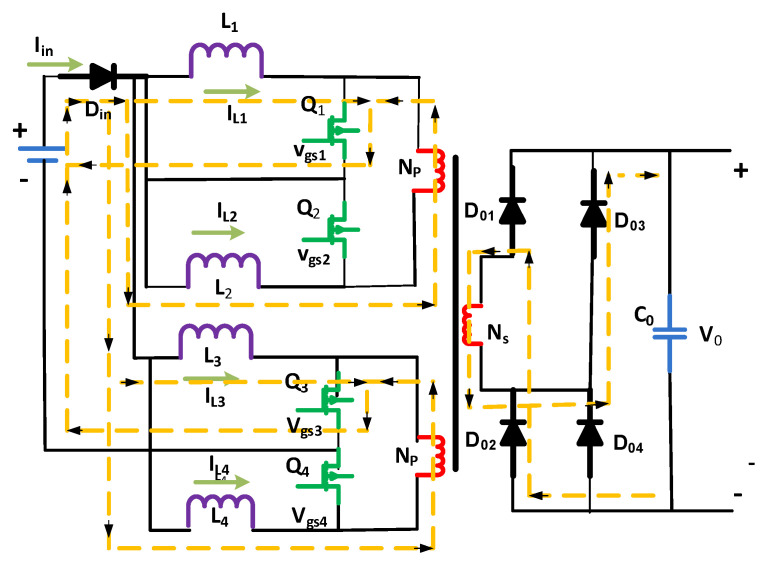
First mode of the circuit.

MOSFETs Q1 and Q3 are turned on in Mode 1, whereas Q2 and Q4 are off. MOSFETs Q1 and Q3 carry the currents inductors $i_{L1}$ and $i_{L3}$, respectively. As Figure 3 depicts, the currents of inductors $i_{L2}$ and $i_{L4}$ drift into the windings of the transformer, i.e., first and second primary, respectively. The following equations give the voltage of inductors $v_{L4}$, $v_{L3}$, $v_{L2}$, and $v_{L1}$ as well as the current of inductors $i_{L4}$, $i_{L3}$, $i_{L2}$, and $i_{L1}$ [28,29,30]:

(3) $$v_{L2}=v_{in}-P_{rimary}$$

(4) $$v_{L4}=v_{in}-n\times V_{0}$$

(5) $$i_{L1}=i_{L1}\left(t_{0}\right)+\frac{\left(v_{in}\times t-t_{0}\right)}{L}$$

(6) $$i_{L\_2}=i_{L2}(t_{0})-\frac{\left(n\times Vo-Vin\right)\times \left(t-t0\right)}{L1}$$

(7) $$i_{L\_3}=i_{L3}\left(t_{0}1\right)-\frac{\left(v_{in}\right)\times \left(t-t_{0}\right)}{L}$$

(8) $$i_{L\_4}=i_{L4}(t_{0}1)-\frac{\left(n\times Vo-Vin\right)\times \left(t-t0\right)}{L}$$

L stands for the inductance of inductors $L_{1}$ to $L_{4}$, and *n* stands for the transformer’s rotation ratio. Lin’s source current is equal to the sum of the inductor currents $i_{L1}$ to $i_{L4}$. This is noted that in the inductors, current $i_{L3}$ and $i_{L1}$ growth is rectilinear. In contrast, the waveforms of inductor currents $i_{L2}$ and $i_{L4}$ reduction are linear when the inductor current behavior is studied in Figure 3. This cancels the source current’s ripple, which is desired, especially for generating lower-voltage and power, e.g., stacked fuel cells and solar arrays. Diodes D03 and D02 transmit the energy towards the load in the case of Mode 1.

### 2.2. Second Mode (t1–t2)

**Figure 4:**
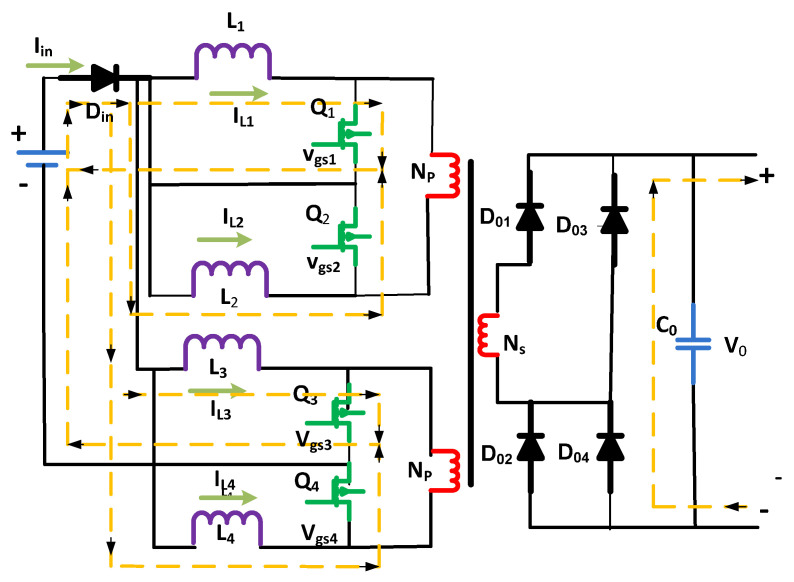
Second mode of the circuit.

The four MOSFETs Q4, Q1, Q2, and Q3 are optimized in Mode 2 as mentioned in Figure 4. Soft switching turns on MOSFETs Q2 and Q4 at time *t*1. The current of the inductors goes down/declines a little before time *t*1. Initially, the inductance leakage transformer tries to oppose the current flow for a while, and the output capacitor of Q2 begins to discharge. As a result, switch Q2 appears to be short-circuited.

The transformer’s leakage inductance is chosen; for example, it opposes the switch current for a short moment so that the draft of the switch approaches its highest rate; or else, Q2 would not switch to zero voltage. The number four, i.e., MOSFET Q4, could also be optimized by applying Zero Voltage Switching (ZVS). To accomplish ZVS, power devices should fulfill the below condition:

(9) $$\frac{I_{in}}{4}\;\geq \frac{nV_{0}}{L_{lk}}\times t_{r}\left(\max.\right)$$

In the above equation, *tr* (max.) shows the maximum time for the rise of the switch’s current. At the same time, $L_{lk}$ shows the transformer’s primary winding leakage. During this time, all electrical switches are turned on.

Similarly, the switches three Q3 and four Q4 of the MOSFETs carry inductor currents $i_{L3}$ and $i_{L4}$, respectively. Inductor currents $i_{L1}$ to $i_{L4}$ grow linearly over this period. The following equations give the voltages of the inductor $v_{L4}$, $v_{L1}$, $v_{L2}$, and $v_{L3}$, as well as the currents of inductors $i_{L4}$, $i_{L3}$, $i_{L2}$, and $i_{L1}$:

(10) $$v_{L1}=v_{in}$$

(11) $$v_{L2}=v_{in}$$

(12) $$v_{L3}=v_{in}$$

(13) $$v_{L4}=v_{in}$$

(14) $$i_{L1}=i_{L1}\left(t_{1}\right)+\frac{\left(v_{in}\times t-t_{1}\right)}{L}$$

(15) $$i_{L2}=i_{L2}(t_{1})-\frac{\left(n\times Vo-Vin\right)\times \left(t-t1\right)}{L}$$

(16) $$i_{L3}=i_{L3}\left(t_{1}\right)-\frac{\left(v_{in}\right)\times \left(t-t_{1}\right)}{L}$$

(17) $$i_{L4}=i_{L4}(t_{1})-\frac{\left(n\times Vo-Vin\right)\times \left(t-t1\right)}{L}$$

Through the transformer, the stored energy is transferred towards the load using the first leakage inductances of the windings. The current (secondary) of the windings of the transformer flops to (0) zero after the stored energy is fully delivered. At this stage, the output of the diodes is disabled. The load is fed by the discharge of the output capacitor Co. The voltages that appear crosswise the two transformer’s primary winding stay at (0) zero during this overlapping time (*t*1–*t*2). Zero voltage switching occurs once the switches Q3 and Q1 are switched off at the moment interval *t*2.

### 2.3. Third Mode (t2–t3)

**Figure 5:**
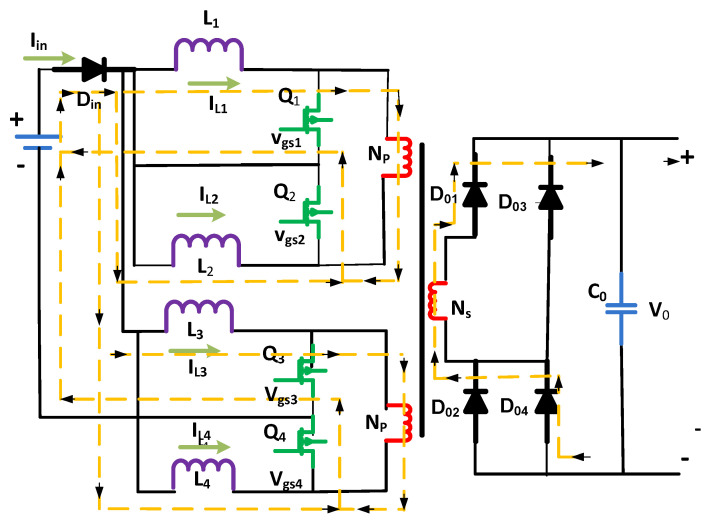
Third mode of the circuit.

MOSFETs Q1 and Q3 are switched off in this mode, whereas Q2 and Q4 are turned on. $i_{L2}$ and $i_{L4}$ inductor currents run through Q2 and Q4, respectively (Figure 5). The first and second primary transformer windings carry inductor currents $i_{L1}$ and $i_{L3}$. The voltages of the inductors $v_{L4}$, $v_{L1}$, $v_{L2}$, and $v_{L3}$, as well as the currents of the inductors $i_{L4}$, $i_{L2}$, $i_{L1}$, and $i_{L3}$, are explained by the Equations during (*t*2–*t*3) [31,32,33,34]:

(18) $$v_{L1}=v_{in}-P_{rimary}$$

(19) $$v_{L2}=v_{in}$$

(20) $$v_{L3}=v_{in}-n\times V_{0}$$

(21) $$v_{L4}=v_{in}$$

(22) $$i_{L1}=i_{L1}\left(t_{2}\right)+\frac{\left(v_{in}\times t-t_{2}\right)}{L}$$

(23) $$i_{L2}=i_{L2}(t_{2})-\frac{\left(n\times Vo-Vin\right)\times \left(t-t2\right)}{L}$$

(24) $$i_{L3}=i_{L3}\left(t_{2}\right)-\frac{\left(v_{in}\right)\times \left(t-t_{2}\right)}{L}$$

(25) $$i_{L4}=i_{L4}(t_{2})-\frac{\left(n\times Vo-Vin\right)\times \left(t-t2\right)}{L}$$

These equations reveal that throughout this interval, the currents of the inductors $i_{L3}$ and $i_{L1}$ reduce, while the currents of the inductors $i_{L4}$ and $i_{L3}$ grow smoothly. As a result, the source current’s ripple is canceled. During this time, the rectifying diodes D4 and D1 transport energy to the load from the source.

### 2.4. Fourth Mode (t3–t4)

**Figure 6:**
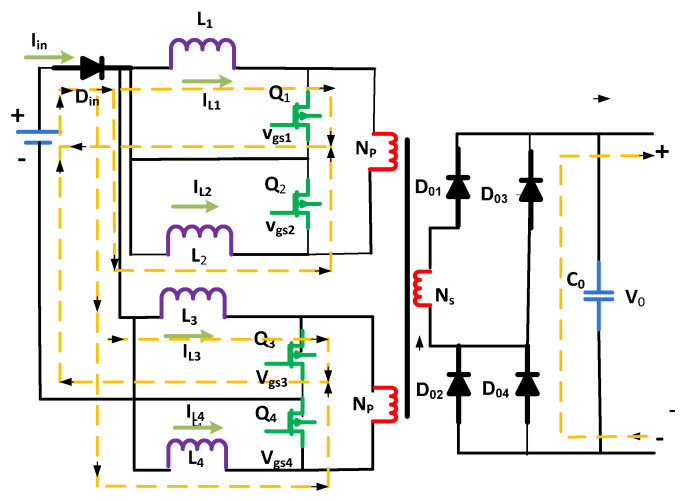
Fourth mode of the circuit.

The four MOSFETs, Q4, Q2, Q3, and Q1, will be kept in on state in this mode as discussed in Figure 6. Soft switching is used to turn on MOSFETs Q1 and Q3. The operation of the circuit is identical to that of Mode 2. The inductor currents $i_{L1}$ to $i_{L4}$ rise in a linear fashion. The following equations give the voltage of the inductors $v_{L4}$, $v_{L2}$, $v_{L3}$, and $v_{L1}$, as well as the current of the inductor $i_{L4}$, $i_{L2}$, $i_{L3}$, and $i_{L1}$ [35]:

(26) $$v_{L1}=v_{in}$$

(27) $$v_{L2}=v_{in}$$

(28) $$v_{L3}=v_{in}$$

(29) $$v_{L4}=v_{in}$$

(30) $$i_{L1}=i_{L1}\left(t_{4}\right)+\frac{\left(v_{in}\times t-t_{4}\right)}{L}$$

(31) $$i_{L2}=i_{L2}(t_{4})-\frac{\left(n\times Vo-Vin\right)\times \left(t-t4\right)}{L}$$

The leakage inductance stores the energy in the two-primary winding of the transformer. This energy is then transmitted from the *Tx* to the load. The current that is called the secondary current of the *Tx* flops to “0” when the overall of the stored energy is delivered. Altogether, the diodes called the secondary rectifying diodes are disabled, and the output capacitor is used only to feed the load. The voltages that appear crosswise the two-primary winding of the transformer continue to be zero over the period (*t*3–*t*4). Soft switching is achieved when MOSFETs Q2 and Q4 turn off at time *t*4. After one interval, *Ts*, the circuit changes back to Mode 1.

## 3. Current and Voltage Stresses of the Switches

The silicon area is reduced when overall switch stress is minimized. During Mode 1 and Mode 3, the peak voltage across switches is identical to the actual voltage of the transformer, which is the reflection of the voltage of the load. The stress can be expressed [36,37,38,39,40] as:

(32) $$V_{stress}=nV_{0}$$

During Mode 1 and Mode 3, the maximum current through a switch is given by:

(33) $$I_{Stress}=I_{L1}=I_{L2}=I_{L3}=I_{L4}=\frac{I_{in}}{2}$$

As a result, the switches only carry 50 percent of the current source. Additionally, actually, it is especially essential in the case of the sources while dealing with lower voltages, for example, stacked fuel cells and modules of solar arrays. Normally, lower voltage sources deliver greater currents in the applications of greater power that necessitate high-current switches. The switch stress is calculated as follows:

(34) $$S=V_{Stress}\times I_{Stress}$$

It is a good idea to compare the potential converter’s overall switch stress and switch utilization. The voltages and currents put on semiconductor devices should be kept to a minimum in a good design. The following is how the total switch stress is calculated:

(35) $$S=\overset{k}{\underset{i=1}{\sum }}V_{i}I_{i}$$

The k represents the actual number of devices (semiconductors). Additionally, the stress of the voltage of the first switch is defined by $V_{i}$, and $I_{i}$ represents the Root Mean Square (RMS) value of the current of the first switch. The maximum amplitude of the wind is frequently referred to rather than the current RMS value. The overall stress of the switch stress can be calculated as follows:

(36) $$S=4\left(nV_{0}\times \frac{I_{in}}{2}\right)$$

The switch utilization is defined as follows:

(37) $$U=\frac{P_{load}}{S}$$

Switch use is kept high in a good converter design.

The efficiency and MOSFET/HEMT stresses of the converters are included in the simulation results. The parameters that are selected for designing the converters are specified below in Table 1.

The transformer’s turn ratio of Flyback boost converter is computed as follows:

(38) $$n=\frac{V_{in}}{V_{0}\times \left(1-D\right)}=0.4$$

Because there are two unknowns in the preceding equation, and it is essential to select a sufficient number for the other unknown before determining the value of the first, *D* = 0.684110 was used to permit overlap among the switches. The computation of the switches’ current and voltage are presented below:

(39) $$V_{Stress}=n\times V_{0}=152\;\mathrm{Volt}$$

(40) $$I_{Stress}=\frac{I_{m}}{2}=10.415\;A$$

The peak-to-peak ripple has been set at thirty percent of the current of the inductor. The inductors inductances are determined in the following manner.

(41) $$L=\frac{DV_{m}}{\Delta i_{L}\times f_{s}}=0.14894\times 10^{3}\;H$$

(42) L≅210 uH

The voltage ripple on the capacitor is set to 0.1 percent of the output voltage. The following is the value of capacitance $C_{0}$ when the ripple voltage is this:

(43) $$C_{0}=\frac{I_{0}\times \left(1-D\right)T_{s}}{\Delta V_{0}}=21.85\;uF$$

The design parameters for the boost converter were chosen based on the supplied parameters. The detailed calculation of the duty ratio for a boost converter is presented here.

(44) $$D=1-\frac{V_{in}}{V_{0}}=0.874$$

The switches’ voltage and current ratings are as follows:

(45) $$V_{Stress}=V_{0}=380\;\mathrm{Volt}$$

(46) $$I_{Stress}=I_{m}=20.83\;A$$

Assume that the maximum ripples, denoted by $i_{L}$, are thirty percent of the current of the inductor. The inductance L is then determined as

(47) $$L=\frac{V_{in}\times DT}{\Delta i_{L}}=67\;uH\times 10^{3\;}H$$

Allow 0.1 percent of the output voltage for capacitor voltage ripple. The output capacitor’s capacitance is then calculated as

(48) $$C=\frac{V_{0}\times DT}{\Delta v\times R}=60.48\;uF$$

## 4. Results and Discussions

**Figure 7:**
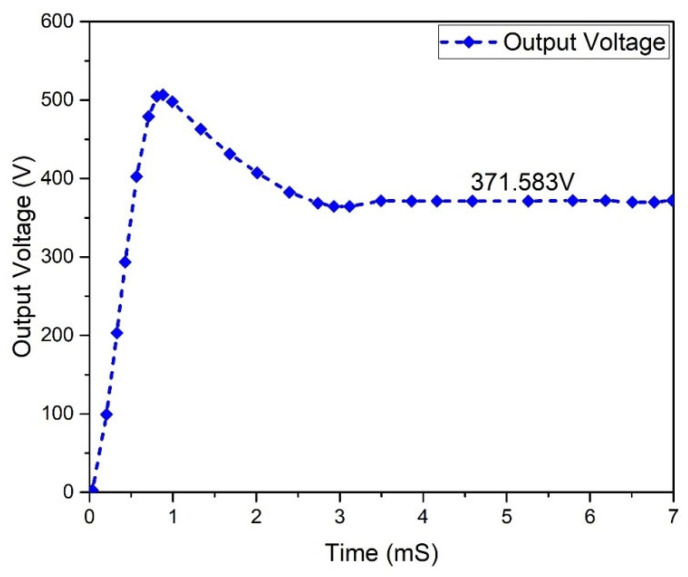
Flyback boost converter output voltage.

Flyback boost, simple boost, and interleaved boost converters were used for low-voltage, high-power renewable energy systems. These converters offer high step-up conversion ratios, high efficiencies, and low switch voltage stresses. They may produce a high output voltage from common voltage sources such as solar arrays and fuel cells. The output voltages and powers waveforms and switch currents and voltages waveforms of the three converters were obtained after successful converter circuit simulation in OrCAD PSpice. The goal was to prove which converter is superior for low-voltage high-power applications in terms of voltage conversion ratio, efficiency, and switch stresses. At the end of the article, Table 1 and Table 2 summarize the simulated converters. Figure 7 depicts the output voltage waveform of the proposed Flyback boost converter. This waveform shows a voltage output of 371.583 V. 

**Figure 8:**
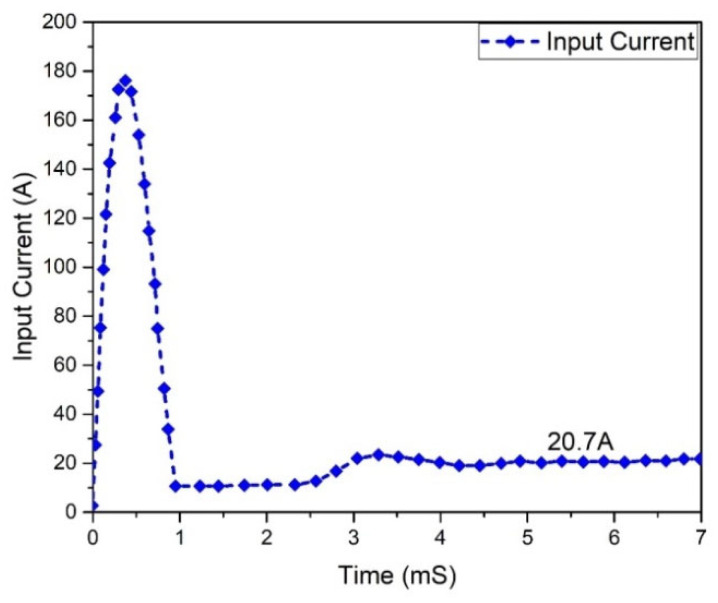
Input current analysis of Flyback boost converter.

In two switching periods, the current of the inductor’s behavior is studied concerning the gate signals of switches S2 and S1. It is the same current flowing through L1 or L3. It illustrates that after the switch S3/S1 is turned on, the current $i_{L1}/i_{L3}$ increases and declines until S1/S3 are turned off again. The inductor current through L2 or L4 was studied, and it was found that its magnitude is the same as the current in L3 or L1. In two switching periods, the currents of inductors $i_{L4}/i_{L2}$ were studied concerning the gate signals of switches S2 and S1. It was found that after the switch S2/S4 is made on, the current $i_{L2}/i_{L4}$ increases and then declines until S2/S4 is turned on again. The waveform/behavior of the input current of the Flyback boost converter is depicted in Figure 8 and reveals a current of 20.7 A. In one switching period, the input current and the inductor currents were studied and demonstrated how the input current ripple is decreased.

**Figure 9:**
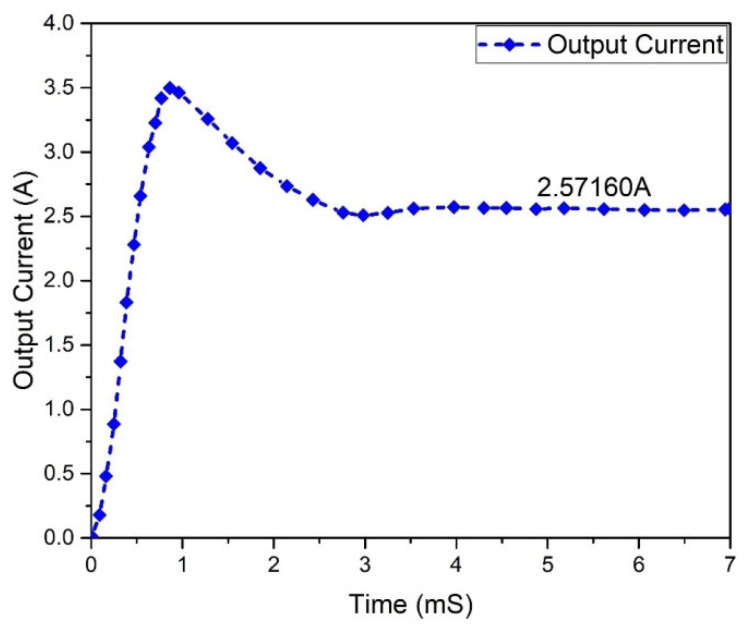
Flyback boost converter output current.

The current of the Flyback boost converter that flows across the load is 2.57160 A, as shown in Figure 9. During one switching interval, the load current was also studied. The load, 70% current, was caused by energy delivered to the load via the transformer and through a pair of diodes. The discharge of the capacitor causes the current of the load via the load during the overlapping period. Since both MOSFETs in each current-fed input circuit were conducting throughout the overlapping period, there was no voltage across the transformer windings because no current was flowing through the transformer’s primary windings.

**Figure 10:**
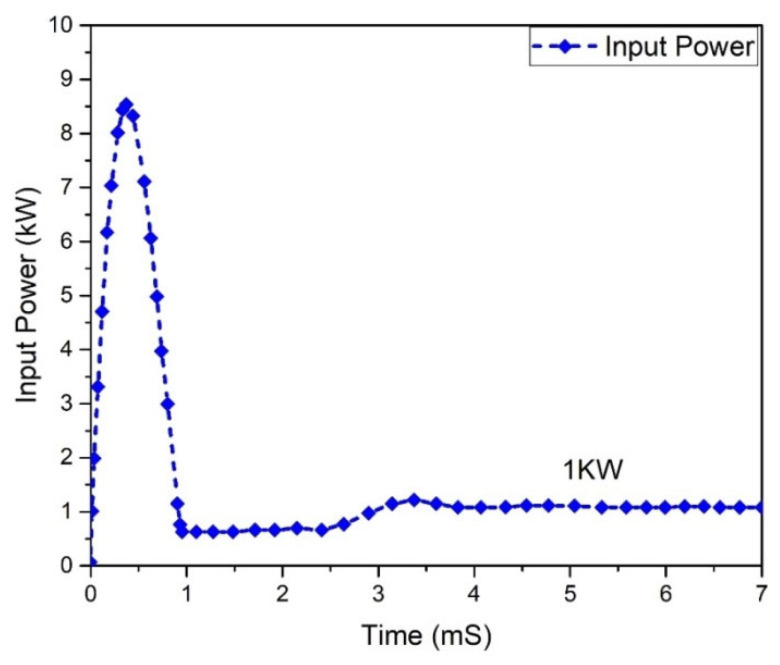
Input power analysis of Flyback boost converter.

**Figure 11:**
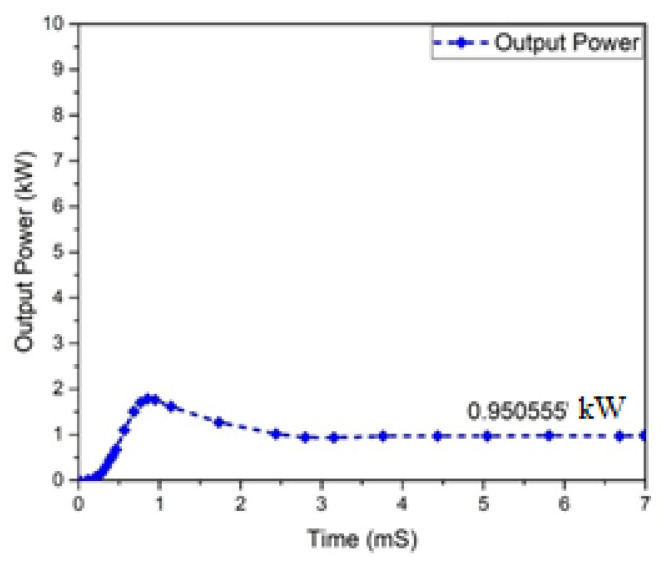
Output power of Flyback boost converter.

Input and output power graphs are shown in Figure 10 and Figure 11. The graph behavior illustrates that the value of the output power was almost 0.950555 W, while the input power value was 1 kW.

**Figure 12:**
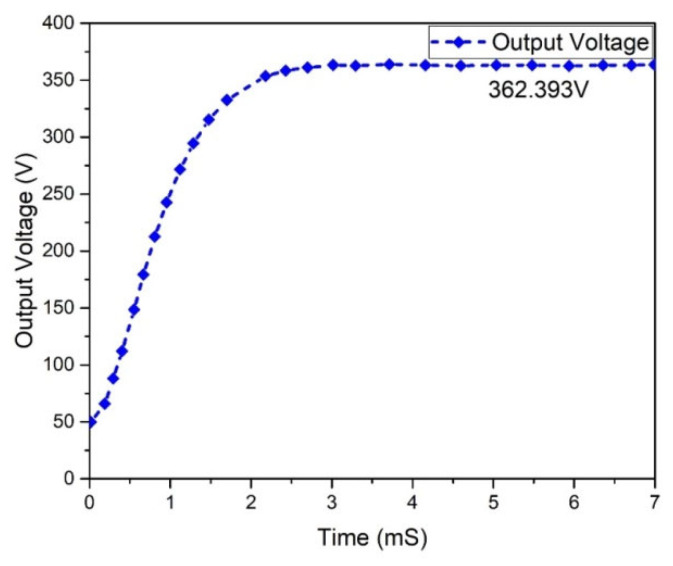
Boost converter output voltage.

**Figure 13:**
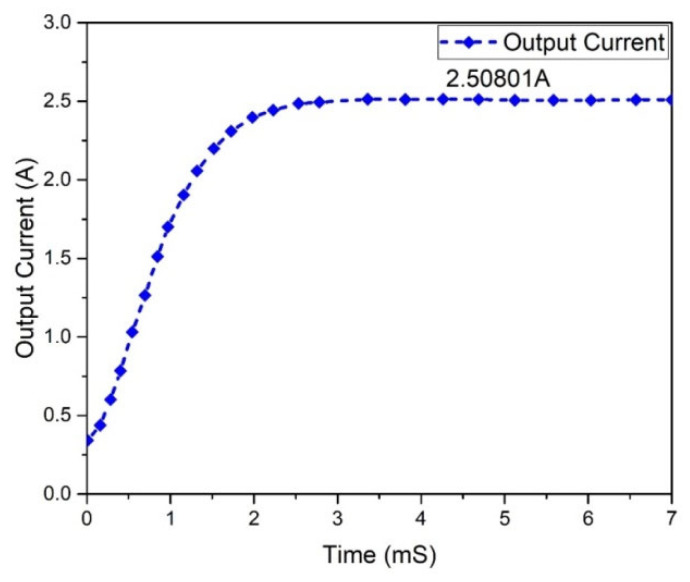
Output current of presented boost converter.

**Figure 14:**
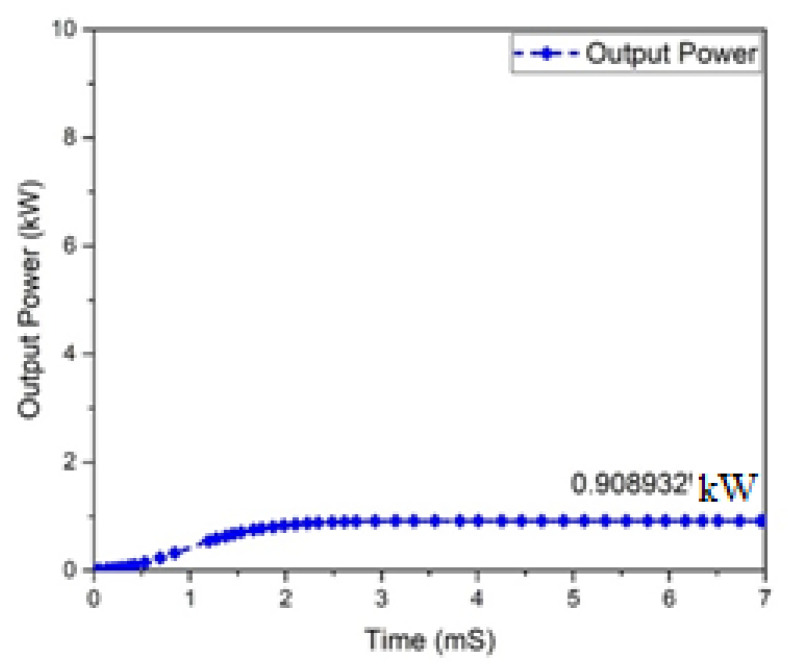
Output power of proposed boost converter.

**Figure 15:**
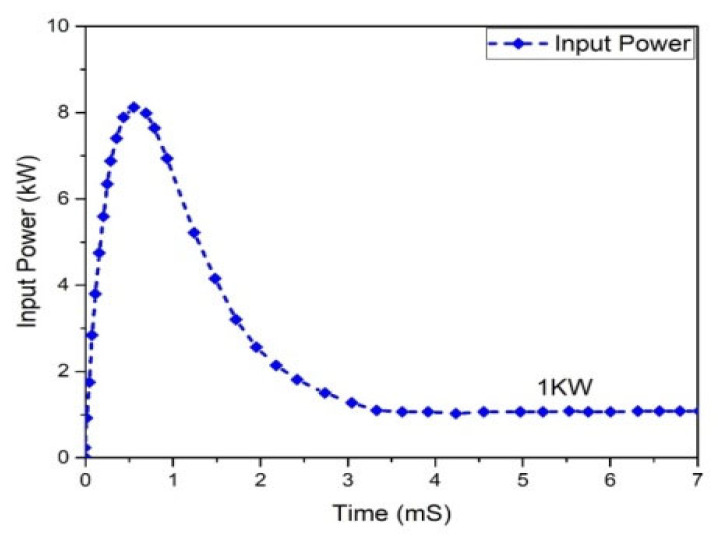
Boost converter input power.

The graphs of the current and voltage behavior of the boost converter are examined in Figure 12 and Figure 13, which show the 362.293 V and 2.50801 A output voltage and current, respectively. The output and input power waveforms are revealed in Figure 14 and Figure 15, respectively. Figure 14 shows the output power of 908.932 W, whereas Figure 15 depicts the input power of 1 KW.

**Figure 16:**
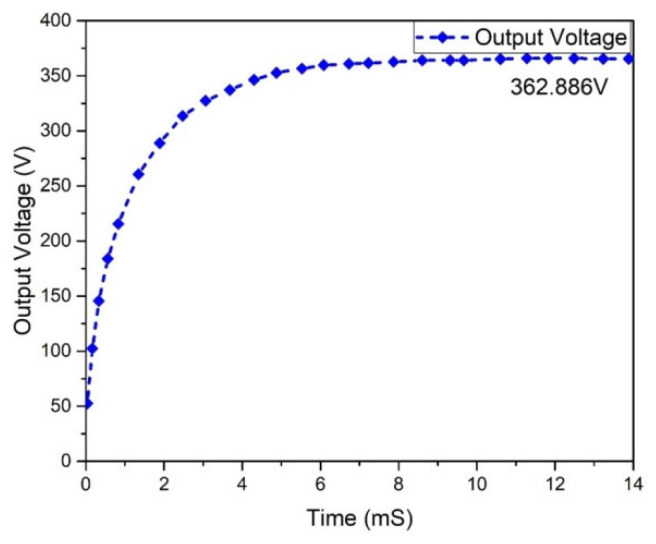
Results analysis of output voltage of interleaved boost converter.

**Figure 17:**
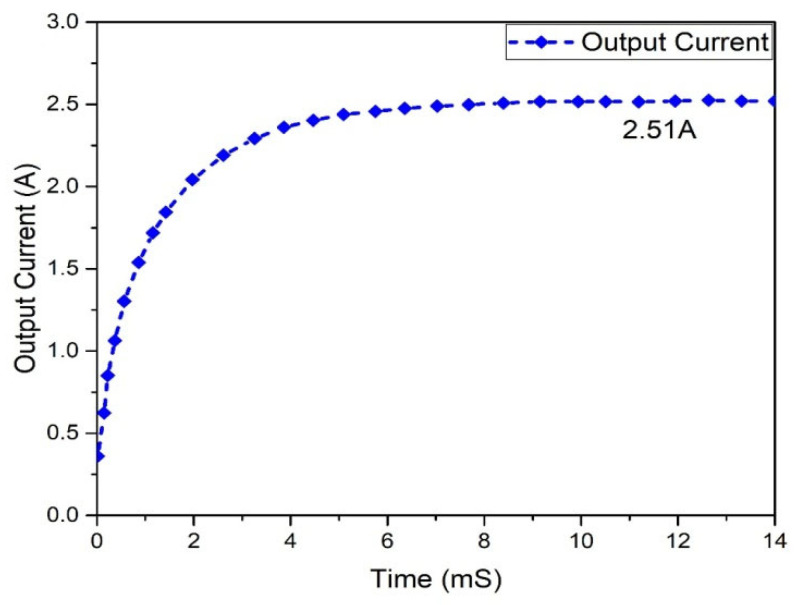
Results analysis of output current of interleaved boost converter.

**Figure 18:**
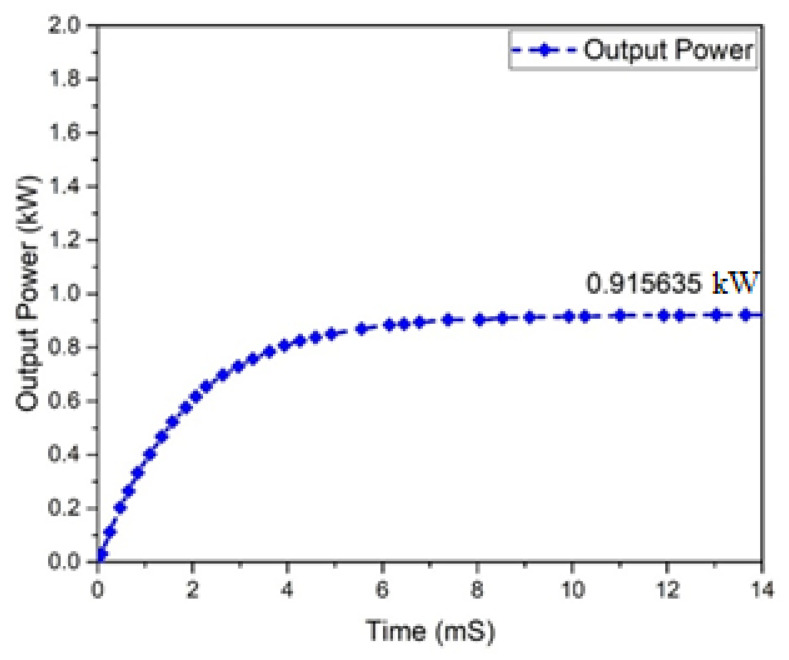
Output power of interleaved boost converter.

**Figure 19:**
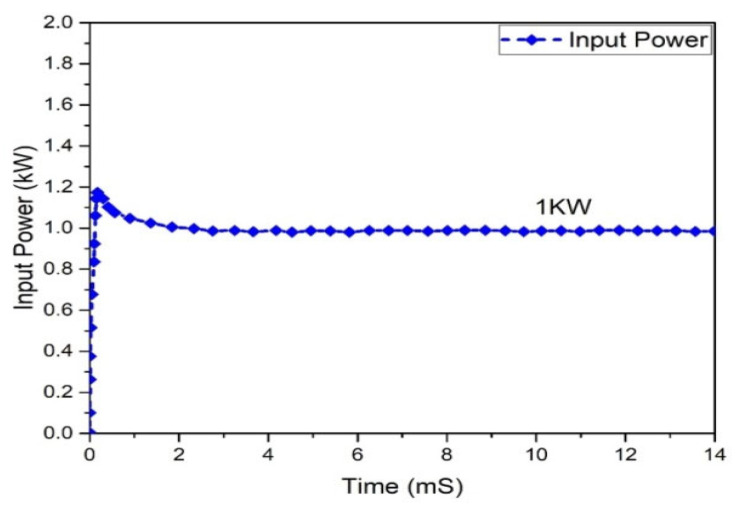
Input power analysis of interleaved boost converter.

Figure 16 depicts the output voltage performance of the suggested interleaved boost converter, which had a value of 362.886 V. The graph of the output current behavior of an interleaved boost converter is revealed in Figure 17, which shows a current of 2.51 A, while the estimated wind was 2.63 A. The input and output power waveforms of the interleaved boost converter are declared in Figure 18 and Figure 19, which exhibit a 911.403 W input power and a 1 KW output power, respectively.

**Figure 20:**
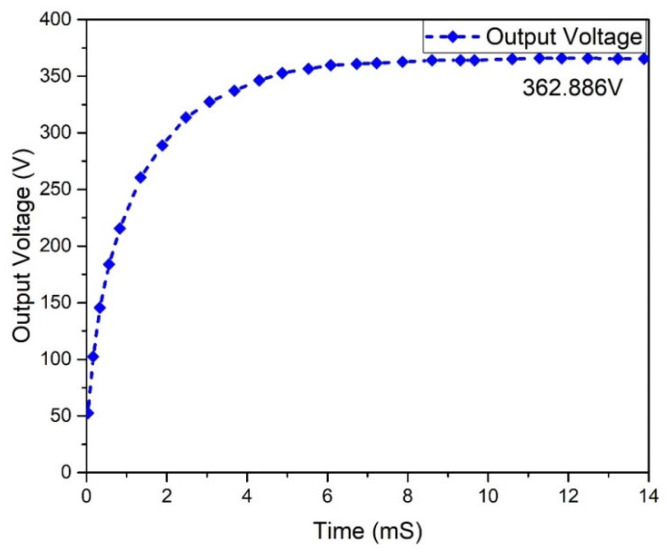
Output voltage of presented Flyback boost converter based (GaN-HEMT).

**Figure 21:**
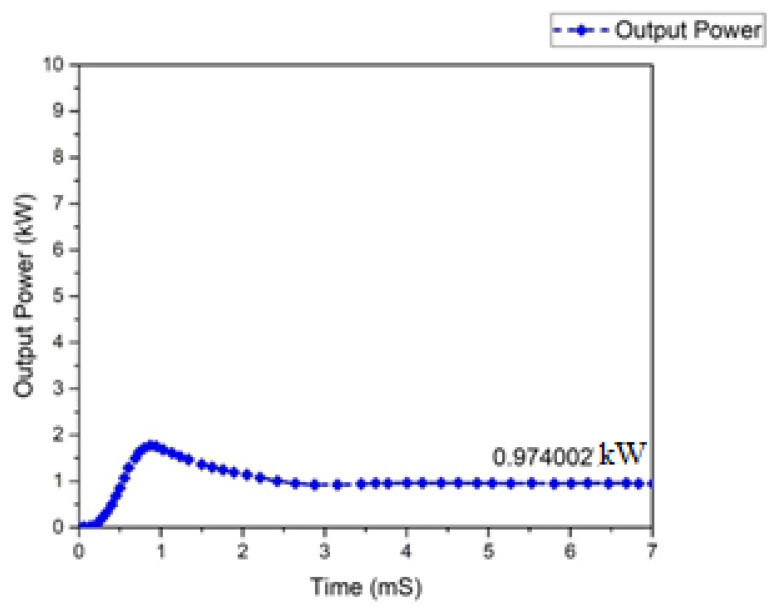
Output power of Flyback boost converter based (GaN-HEMT).

**Figure 22:**
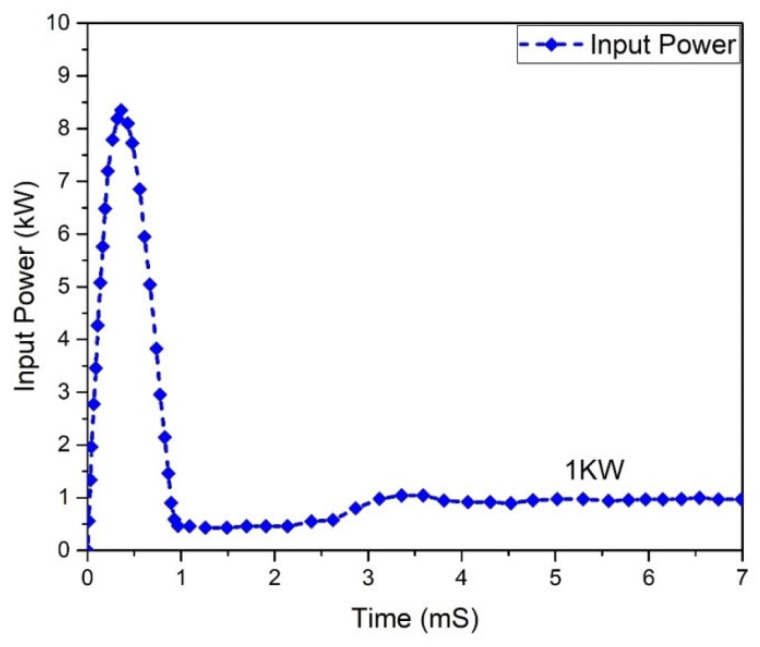
Input power of Flyback boost converter (GaN-HEMT).

The experimental analysis of the Flyback-Based converter (GaN HEMT) is presented in Figure 20, which reveals the behavior of the output voltage. Figure 21 and Figure 22 illustrate the circuit’s power based on GaN-HEMT after the voltage is raised using a Flyback boost converter.

**Figure 23:**
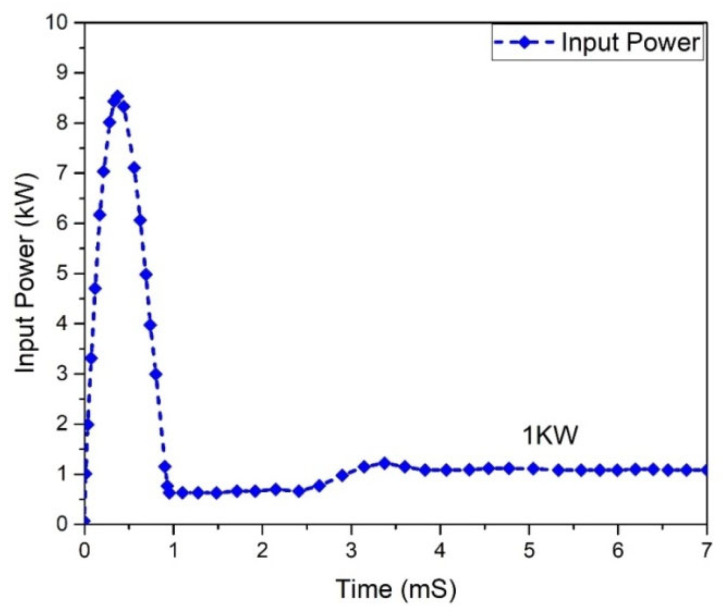
Input power of Flyback boost converter (Si-MOSFET).

**Figure 24:**
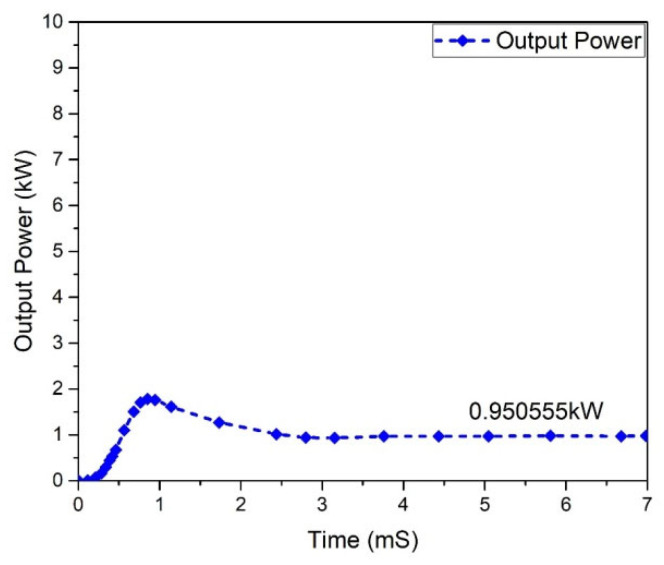
Output Power of Flyback boost converter (Si-MOSFET).

One of the most significant characteristics of any converter is its efficiency. All power-processing applications necessitate high-efficiency converters. It is unfeasible to construct a converter if it demonstrates a lower efficiency. In a steady condition, the waveforms of the input power and output of the Flyback boost converter can be seen in Figure 23 and Figure 24. As indicated, the value of the input power was 1 kW. In comparison, the output power was raised to 0.950555 kW for the mentioned input. 

The Flyback-based converter’s efficiency is estimated as

(49) $$\eta =\frac{Output\;Power}{Input\;Power}=0.950555=95\%$$

**Figure 25:**
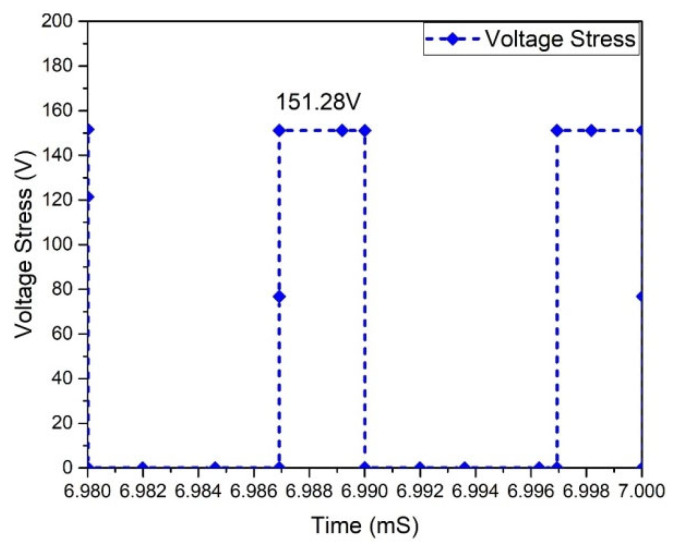
Voltage stress of MOSFETs.

**Figure 26:**
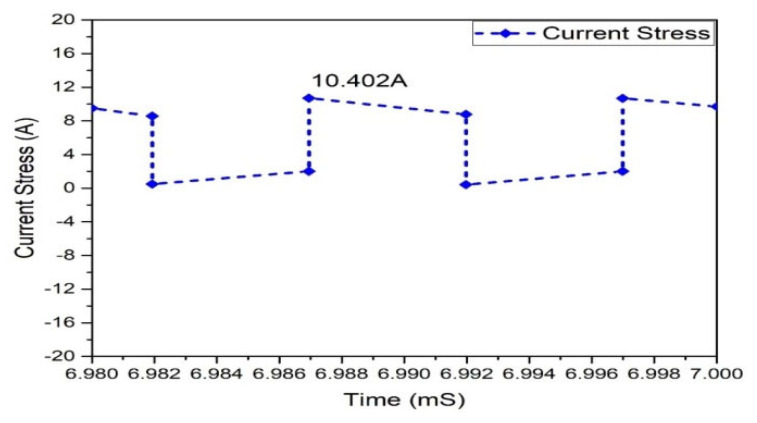
Current stress of MOSFETs.

At this stage of operation, Modes 3 and 1 are observed, and it has been found that in the case of the Flyback boost converter, the value of the peak voltage crosswise the MOSFET appears. The MOSFETs used in the converter must tolerate the high peak voltages. MOSFET voltage waveforms are shown in Figure 25. The Flyback-based converter represents the voltage stress of 151.284 V across all MOSFETs. In a Flyback-based converter, the maximum current via a MOSFET occurs in Modes 1 and 3. This is significant because the MOSFETs used in the converter must withstand the maximum current stress. Current stress waveforms in MOSFETs are shown in Figure 26. It shows that each MOSFET has the current stress of 10.310 A.

**Figure 27:**
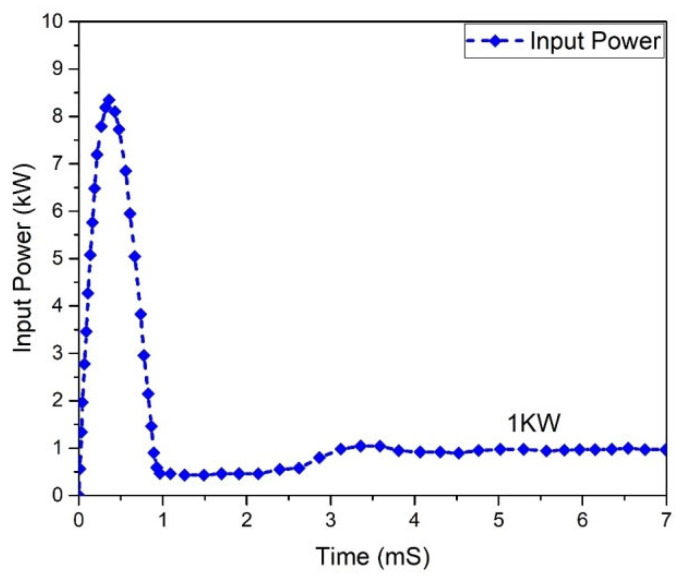
Input power (GaN-HEMT).

**Figure 28:**
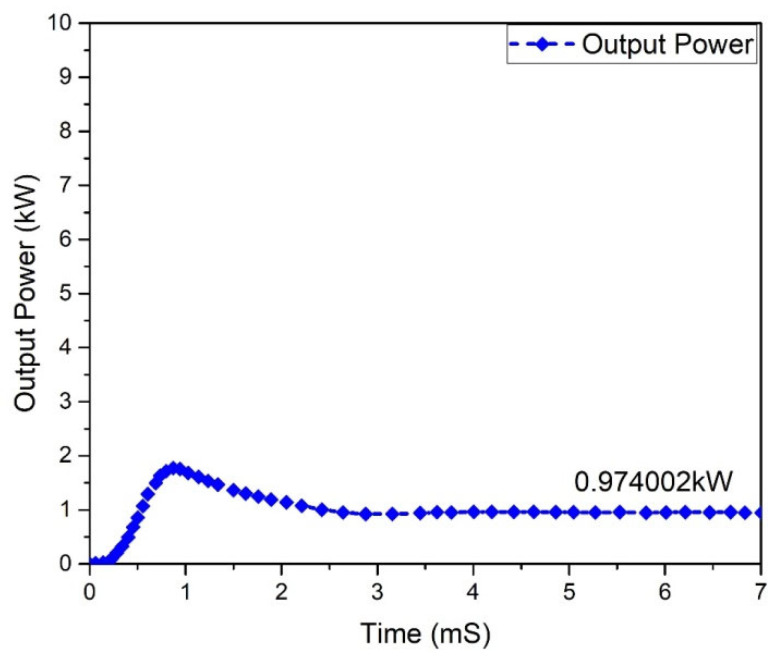
Output power (GaN-HEMT).

All power-processing applications necessitate the use of high-efficiency converters. When a converter’s efficiency is low, creating one is impractical. The graphs of the input and output power behavior for a (GaN HEMT) based Flyback-based converter are shown in Figure 27 and Figure 28. According to the table below, the input power value is noted to be 1.0 kW, and the value of the power at the output is 974.002 W.

The Flyback-based converter based (GaN-HEMT) has the following efficiency:

(50) $$\eta =\frac{Output\;Power}{Input\;Power}=0.974002=97\%\;$$

**Figure 29:**
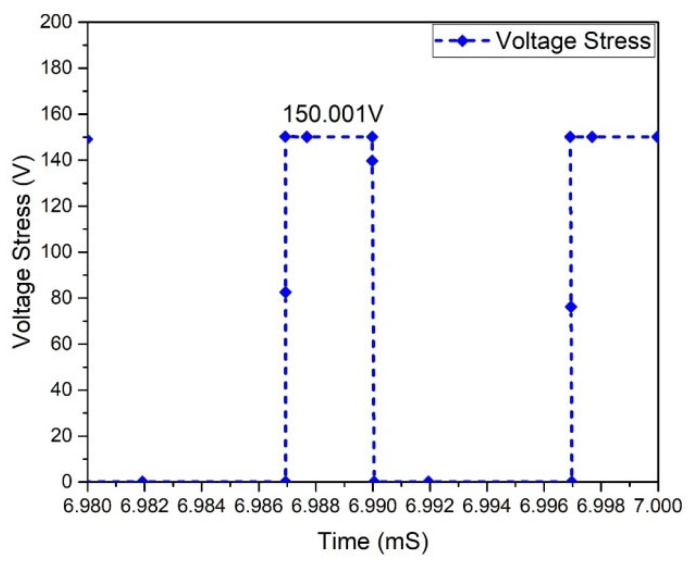
Voltage stress of HEMT.

In the operational stage in Modes 1 and 3 of a Flyback-boost converter, the maximum voltage is passed across the HEMT; thus, the HEMTs converters must be able to tolerate peak voltages. Voltage waveforms of HEMTs are shown in Figure 29, which offers a voltage stress of 150 V across all HEMTs in the Flyback-based converter.

**Figure 30:**
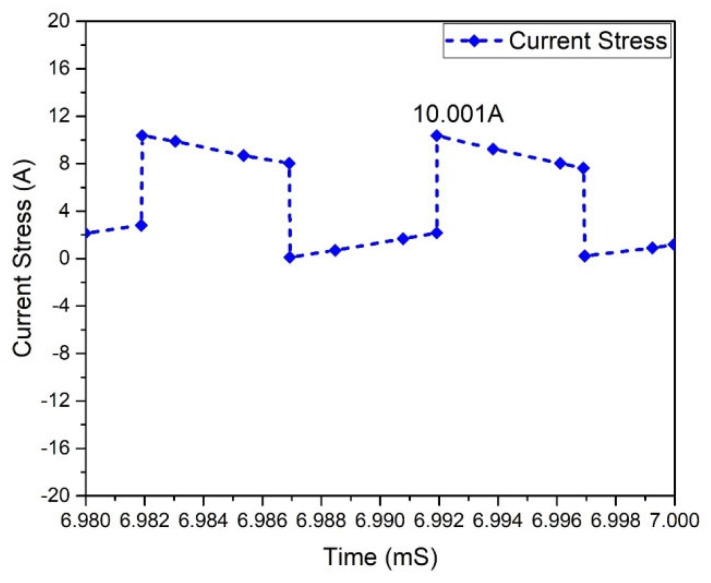
Current stress of HEMT.

In a Flyback-based (GaN-HEMT) boost converter, the maximum current via a HEMT occurs in Modes 1 and 3. This is significant because the HEMT used in the converter must withstand the maximum current stress. Waveforms of current strains in HEMT are shown in Figure 30. The current stress in each HEMT is 10 A, according to the data.

Table 2 explains the relations among the Flyback Si-MOSFET converter, boost Si-MOSFET converter, interleaved boost Si-MOSFET converter, and Flyback boost GaN-HEMT in terms of efficiency, voltage stress, and current stress. This shows that the efficiencies of the proposed Flyback boost GaN-HEMT converter and the Flyback Si-MOSFT converter are better than current approached. The correlation of the presented work with the current system is depicted in Table 3, which explains the optimum solutions of the proposed system as compared to current models. 

## 5. Conclusions

In this study, three DC–DC converters were investigated for a higher power and lower voltages in power systems based on renewable energy: Flyback-based converter, Interleaved boost converter, and standard Boost converter. In a system that is a combination of the tacked fuel cells and PV arrays modules, the low voltages are boosted to 380.00 V, and the nature of voltage is DC, which is essential for a complete bridge inverter for the creation of 220 V having the nature of AC to be interfaced to the 220.00 V AC grid. Utilizing the setup of PSpice, the converters have been developed. The simulation has been carried out, selecting the proposed technologies to analyze the selection of the best technology and model. High-voltage conversion ratios are used in the converters. Additionally, the only better circuit to be used is to improve the production quality and thus the efficiency of the renewable energy systems. That has dramatically brought a change. It has become possible because of the availability of the converters consisting of a high efficiency and connecting the resources of renewable energies to the grid-connected power system running on 220 V AC. Consumer demand for electricity varies greatly. When a renewable energy system is connected directly to consumers rather than through a grid, it is not easy to operate properly. Because of the fluctuating nature of consumer load, this is the case. According to the simulation results, the Flyback-based converter is the best choice for connecting stacked fuel cell and PV arrays modules (lower voltages) to the grid of 220.00 V AC. In this case, the main component used is the inverter that converts DC to AC. In short, the Flyback-based converter has the best topology with a higher efficiency, minimal stress of the device, and higher switching frequency. Additionally, GaN-based technology is the most effective way to boost converter efficiency.

In the present research work, the two technologies, i.e., Si-MOSFET- and GaN-HEMT-based converters, were studied. This study can be extended by considering SiC-MOSFET-based converters. The proposed GaN-HEMT-based boost converters can be further investigated for electric and hybrid vehicles.

## Figures and Tables

**Table 1:** Parameters detail used for design converters.

Parameter	Description
Input power	1 kW
Input voltage	48 V
Input current	20.83 Amp
Output voltage	380 volts (for full bridge inverter)
Output current	2.63 Amp
Resistance	144.487 Ohm

**Table 2:** Characteristics of Flyback boost, simple boost, and interleaved boost converters Si-MOSFET/GaN-HEMT.

S.NO	Parameter	Flyback-Based Converter (Si-MOSFET) (%)	Boost Converter (Si-MOSFET)	Interleaved Boost Converter (Si-MOSFET)	Flyback Boost Converter (GaN-HEMT)
1	Efficiency	95	93.5	91	97
2	Voltage Stress	151.28	364.68	362.98	150.001
3	Current Stress	10.402	26.02	7.6327	10.001

**Table 3:** Comparison of the proposed model with current approaches.

Description	[40]	[41]	[42]	This Work
Efficiency	95	94	95.5	97
Voltage stress (V)	380	380	376	152
Current stress (A)	20	19.5	19	11
Voltage gain (G)	(3-d1-d2)/(1-d1-d2)	4/1-d1	4/1-d1	D1/1-D1
Diodes	4	3	3	2
Switches	4	3	2	2
Inductors	3	4	3	2
Capacitors	4	3	2	1

## Data Availability

The data is available in the paper.
